# Effects of Neuromuscular Electrical Stimulation Synchronized with Chewing Exercises on Bite Force and Masseter Muscle Thickness in Community-Dwelling Older Adults in South Korea: A Randomized Controlled Trial

**DOI:** 10.3390/ijerph17134902

**Published:** 2020-07-07

**Authors:** Ji-Su Park, Young-Jin Jung, Min-Ji Kim

**Affiliations:** 1 Advanced Human Resource Development Project Group for Health Care in Aging Friendly Industry, Dongseo University, Busan 47011, Korea; jisu627@hanmail.net; 2 Department of Radiological Science at Health Sciences Division, DongSeo University, Busan 47011, Korea; 3 Department of Dental Hygiene, DongSeo University, Busan 47011, Korea

**Keywords:** electrical stimulation, masseter muscle, mastication, occlusal force, resistance training, sarcopenia

## Abstract

This study is aimed at investigating the effects of synchronized neuromuscular electrical stimulation (NMES) and chewing exercises on bite force and the masseter muscle thickness in community-dwelling older adults. *Material and methods:* Forty older adults were enrolled in South Korea and randomly assigned to either an experimental or control group. The experimental group performed chewing exercises using the No-Sick Exerciser equipment synchronized with NMES applied to the bilateral masseter muscles, while the control group performed only chewing exercises. Both groups received interventions for 20 min/day, 5 days/week, for 6 weeks. Bite force was measured using the OCCLUZER device, and masseter muscle thickness was measured using a portable ultrasound. *Results:* Both groups showed a significant increase in bite force and masseter muscle thickness compared to baseline measurements (*p* < 0.05). The experimental group showed a significantly higher increase in bite force and masseter muscle thickness than the control group after combined intervention (*p* < 0.05). *Conclusion:* This study demonstrates that NMES synchronized with chewing exercises is more efficient in increasing bite force and masseter muscle thickness than chewing exercises alone in community-dwelling older adults.

## 1. Introduction

The masticatory muscles are involved in chewing food and forming a bolus in the oral phase of the swallowing process [1,2]. They consist of four muscles: the masseter, temporalis, and lateral and medial pterygoid muscles. Of these, the masseter is the primary chewing muscle that produces the most powerful force [3,4].

Sarcopenia is a common problem in aging individuals, characterized by atrophy of the skeletal muscles and weakness [5]. This may include weakness and atrophy of the masseter muscle, which can cause difficulties in the oral phase of swallowing. Reduced chewing function decreases the pleasure of food and may negatively affect nutritional intake, which can lead to various complications, such as dehydration, malnutrition, weight loss, and aspiration pneumonia [6,7,8]. Therefore, chewing exercises are important to ensure safe swallowing in older individuals who are prone to masseter atrophy.

Chewing exercises help to improve masticatory function. As a standard part of the method, resistance training induces myophysiological changes, such as activation and thickness gain of muscles [9,10,11]. Several studies have reported that chewing exercises improve motor function during chewing and increase the maximum occlusal force in children, older adults, and patients with neurological disorders [2,12,13]. It is hypothesized that the increased activation of the masticatory muscles through resistance training contributes positively to the physiological changes, such as increased muscle thickness and to the maximum occlusal force of the masseter muscle as a result of its repeated use.

Neuromuscular electrical stimulation (NMES) is known as a treatment modality for neuromuscular training that delivers stimulation to the muscles through a surface electrode. It is used in facilitation techniques to increase muscle strength and sensory awareness and to prevent muscle atrophy, thereby improving motor function [14,15]. Recently, several studies have demonstrated that NMES does not only induce the activation of the masticatory muscle [16,17] but is also effective for improving oral functions, including masticatory functions in the oral phase of the swallowing process [18,19], which might be due to cortical excitability and physiological changes induced by NMES.

Other studies reported synergistic effects of NMES combined with voluntary exercise in improving the motor function of skeletal muscle and inducing physiological changes [20,21,22]. The masseter muscle, as a skeletal muscle, is largely composed of two different muscle fiber types that need to be exercised to the same extent [23]. Voluntary exercise primarily stimulates type 1 fibers, whereas NMES stimulates type 2 fibers [21]. Therefore, NMES applied simultaneously with chewing exercises may prove to be more effective in improving masseter muscle motor function than employing voluntary exercise alone. However, there is currently no clinical evidence on the effects of simultaneous chewing exercises and NMES of the masseter muscle in improving bite force and masseter muscle thickness. Therefore, the purpose of this study is to investigate whether NMES synchronized with chewing exercises is more efficient at improving masseter muscle thickness and occlusal force than chewing exercises alone.

## 2. Materials and Methods

### 2.1. Participants

The sample size was calculated using G-Power 3.1 software (University of Dusseldorf, Dusseldorf, Germany). The power and alpha levels were set to 0.80 and 0.05, respectively, and the effect size was set to 0.85 based on the results of the pilot study. As a result, at least 18 people were required for each group, and a total of 40 participants were recruited for this study, considering potential drop-out. Inclusion and exclusion criteria were based on previous studies [24] and are shown in Table 1. Table 2 shows the general characteristics of the participants. Prior to the initiation of this study, written consent was obtained from all participants, and all procedures were approved by the Seoul Medical Center Institutional Review Board (SEOUL 2019-05-005).

### 2.2. Study Design and Setting

This study was conducted using a prospective, two-group, randomized, controlled trial design. The experiment was performed at the Sasnag-Gu Seniors Welfare Center in Busan, South Korea.

### 2.3. Study Procedures

All participants were randomly assigned to either the experimental or the control group. The experimental group underwent NMES synchronized with chewing exercises. NMES was applied using STIMPLUS DP200^®^ (Cybermedic Corp, Iksan, South Korea). The NMES unit provided two channels of bipolar electrical stimulation at a 60 Hz pulse frequency and pulse interval of 500 μs. Two pairs of electrodes were attached to the masseter muscles (on both sides) by an occupational therapist. The intensity of the NMES was gradually increased by 0.5 mA in intervals and, finally, the intensity was increased until the motor level was reached. The intensity of the NMES applied to the experimental group was applied differently according to the participant’s compliance and was set to an average of 7.3 ± 2.5 mA.

**Figure 2:**
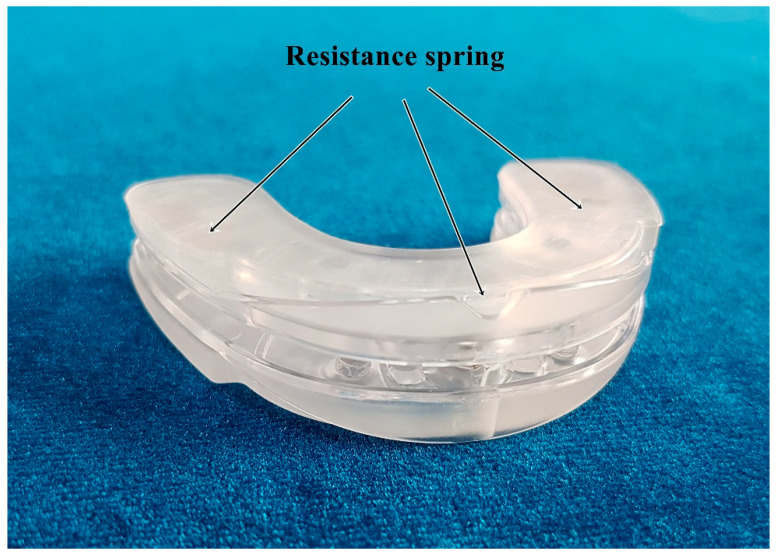
No-Sick Exerciser.

**Figure 3:**
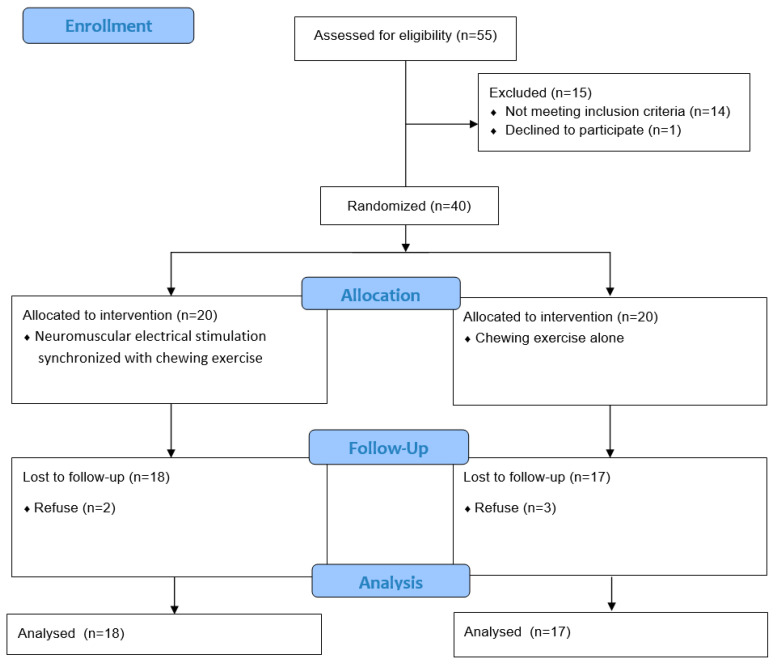
Flowchart of the trial.

The chewing exercises used the No-Sick Exerciser (No-Sick Exerciser, HiFeelWorld Inc., Seoul, South Korea) as a chewing exercise device. The No-Sick Exerciser has a U-shaped frame with small springs that are mounted on both sides and in the center of the unit, which exert resistance to movements during chewing (Figure 2). The device is inserted into the mouth to fit between the upper and lower teeth. In our study, the training was divided into isometric and isotonic exercises. The isotonic training consisted of repeated concentric and eccentric contractions against the resistance of the springs at 2-s intervals. Isometric training consisted of biting down the device and sustaining the bite for 10 s, which was repeated after about 5 s of rest. In the experimental group, simultaneous chewing exercises with the application of NMES were performed 20 min/day, 5 days/week, for 6 weeks (Figure 3).

The control group performed chewing exercises without NMES. The chewing exercises were performed following the same protocol and intervention period as that of the experimental group. A new No-Sick Exerciser was provided to all participants and was washed with water immediately before and after use.

### 2.4. Outcome Measurements

Masseter muscle thickness was measured by an experienced radiologic technician using an ultrasound device (Sonon, Healcerion, Seoul, South Korea). The participants were instructed to sit in contraction during the scan of the left and right masseter muscles. The linear probe was set to a frequency of 10 MHz, 66 dB, and was aligned using the acanthiomeatal line as the baseline for all participants. Subsequently, it was moved caudally by 2–3 cm to correspond with the mouth tail and the midpoint of the zygomatic arch and mandibular angle. Further, the probe was moved cranially by 2–3 cm to correspond with the outer canthus and masseter muscle level. The thickness of the masseter muscle was determined as the thickest part of the image.

Bite force was measured by an experienced radiologic technician using an Occluzer device (ACCURA, Demetec, Gyeonggi-do, South Korea). Measurements were obtained by orienting the Frankfort horizontal plane parallel to the floor. The participants’ mouths were opened slightly and a pressure-sensitive film (disposable pressure film, Gyeonggi-do, South Korea) was inserted into the oral cavity, and the participants were instructed to bite the pressure-sensitive film with as much force as possible. While maintaining a sitting position, the participants were instructed to place their incisors at the midpoint of the bite sensor. Bite force was measured for 5 s, and an average of three measurements was recorded for further analysis. The maximum occlusal force was calculated in Newtons (N). All parameters were blinded to the investigator who performed the assessment of the muscle thickness and measurement of bite force using ultrasound and pressure sensing devices.

### 2.5. Statistical Analysis

All statistical analyses were performed using SPSS version 15.0 (IBM Corporation, Chicago, IL, USA), with the level of significance set at *p* < 0.05. Descriptive statistics are presented as means with standard deviations. A Shapiro–Wilk test was used to verify the normality of the outcome variables. A paired t-test was used for intragroup comparison, and an independent t-test was used for comparison between groups. In addition, this study calculated and interpreted effect sizes (Cohen’s d).

## 3. Results

### 3.1. Participants

Out of the 40 enrolled participants, five were excluded from the study. We analyzed the data of 35 participants. The flowchart of the study is shown in Figure 3.

### 3.2. Effect on Masseter Thickness

Both groups showed a significant improvement in masseter thickness from before to after the intervention (*p* < 0.05). When comparing the results between the two groups after the intervention, the experimental group showed significantly higher values for the masseter thickness than the control group (*p* < 0.001) (Table 3). The amount of change in the experimental group was significantly larger than that in the control group (*p* < 0.001) (Table 4).

### 3.3. Effect on Occlusal Force

Both groups showed a significant improvement in the occlusal force after as compared to before the intervention (*p* < 0.05). When comparing the two groups after the intervention, the experimental group showed significantly higher values for occlusal force than the control group (*p* < 0.001) (Table 3). The amount of change in the experimental group was significantly larger than that in the control group (*p* < 0.001) (Table 4).

Chewing exercises are commonly used in clinical practice as a therapeutic exercise method that can lead to an increase in masseter muscle thickness and bite force through voluntary muscle contraction. Additionally, NMES is a modality that can improve muscle physiology and motor function by evoking contraction of the skeletal muscle. Therefore, these two methods may have the advantage of being applied at the same time, and it is possible to expect a synergistic effect. To the best of our knowledge, this is the first study to investigate the effects of chewing exercises synchronized with NMES on occlusal force and masseter muscle thickness. We demonstrated that chewing exercises synchronized with NMES were more effective than chewing exercises alone for increasing muscle thickness and occlusal force.

The results of this study may be explained by the synergistic effects of chewing exercises and NMES in stimulating the two types of muscle fibers contained in the masseter. Skeletal muscle fibers are divided into type 1 (red muscle, slow-twitch, and long-duration motor unit) and type 2 (white muscle and fast-twitch motor unit) [25]. All skeletal muscles have both type 1 and type 2 fibers, but the proportion of each of type differs between individual muscles. In the masseter muscle, the exact ratio varies depending on a person’s craniofacial form [23].

Chewing is a traditional method of resistance training that causes voluntary muscle contractions in the masseter through exercise, resulting in myophysiological changes, such as muscle thickness and strength. Previous studies have shown that chewing activates the masseter muscle [26], which increases motor unit recruitment to peripheral nerves and consequently contributes to increased muscle thickness and strength [27,28]. Voluntary exercise, such as chewing, recruits predominantly type 1 muscle fibers, which are typically activated by less force than type 2 fibers [29,30], meaning that this exercise is more suitable for stimulating type 1 than type 2 fibers. However, sarcopenia observed with aging affects type 2 fibers more than type 1 [30]; therefore, it is important to stimulate both type 1 and type 2 muscle fibers in older adults.

Conversely, NMES stimulates forceful contractions, especially in type 2 muscle fibers [30,31,32,33,34]. The primary mechanism underlying NMES is that it induces muscle contractions by depolarizing nerve fibers, which helps not only to increase muscle strength and prevent muscle atrophy but also to re-educate the muscle on certain movements and improve circulation [32,33]. During normal muscle contraction, type 1 fibers are recruited before type 2 fibers. Recruitment patterns during NMES are reversed, with type 2 fibers being recruited first [34]. Therefore, theoretically, synchronous recruitment of type 1 and 2 fibers by NMES combined with chewing exercises might provide more effective masseter muscle activation, thereby leading to a more significant therapeutic effect than chewing exercises alone [35,36].

Physiological changes in muscle tissue are closely related to muscle strength and motor function. Increasing muscle thickness suggests an increase in the force that can be generated. In this study, we measured the occlusal force, which depends on the teeth and masseter muscle. We tried to ensure homogeneity of the dental condition of patients through the selection and exclusion criteria. Chewing exercises increase the thickness of the masseter muscle, which directly affects occlusal force. However, our findings suggest that synchronized NMES and chewing exercises induced more myophysiological changes in the masseter, which may have a direct effect on occlusal force.

This study confirmed the synergistic effect of synchronized NMES and chewing exercises, but there may be several factors to consider. The high intensity of chewing and NMES causes sufficient muscle contraction, but the compliance is low in performance because it causes discomfort, temporary pain, and muscle fatigue. However, the low intensity of chewing and NMES makes it difficult to induce sufficient muscle contraction, but, because of higher compliance, it can be performed repeatedly and for a long time. Therefore, it may be necessary to determine these factors based on the patient’s overall medical information, such as physical condition and cooperation.

This study has some limitations. First, the number of participants was small, and the results, hence, cannot be generalized. Second, while the degree of resistance is an important factor in chewing exercise, our equipment did not allow for different degrees of resistance. Third, long-term effects cannot be confirmed because we did not follow patients beyond the end of the intervention period.

## 4. Conclusions

The present study’s results demonstrate that NMES synchronized with chewing exercises effectively improve masseter muscle thickness and occlusal force to a greater extent than chewing exercises alone, but further studies with larger study groups would be necessary to validate our results.

## Figures and Tables

**Figure 1:**
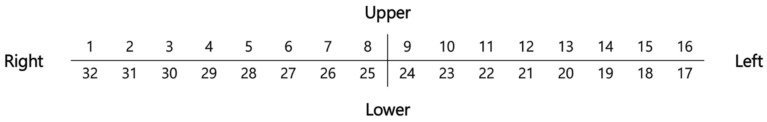
Universal numbering system of teeth.

**Table 1:** Inclusion and exclusion criteria.

Inclusion Criteria	Exclusion Criteria
-No reported history of neurological diseases (e.g., stroke, dementia)	-Significant malocclusion or facial asymmetry
-Normal speech and swallowing function	-Orofacial pain (e.g., trigeminal neuropathy and toothache)
-Normal oral structure	-Periodontal disease
-No periodontal disease	-Parafunctional oral habits (e.g., finger sucking, lip-, cheek- and object-biting, bruxism, and nail-biting)
-Ability to perform activities of daily living without the help of a caregiver	-Parafunctional oral habits (e.g., finger sucking, lip-, cheek- and object-biting, bruxism, and nail-biting)
-Ability to communicate and cooperate	-Diagnosis of obesity
-At least 20 teeth remaining (depending on the universal numbering system, at least five teeth remaining from 1, 2, 3, 4, 5, 6, 7, 8; at least five teeth remaining from 9, 10, 11, 12, 13, 14, 15, 16; at least five teeth remaining from 17, 18, 19, 20, 21, 22, 23, 24; or at least five teeth remaining from 25, 26, 27, 28, 29, 30, 31, 32) (Figure 1)	-Regular gym visits within six months prior to enrollment

**Table 2:** A summary of the clinical and demographic features of the subjects.

	Experimental Group	Control Group
Number of subjects	18	17
Gender (male/female)	8/10	8/9
Age (year)	74.1 ± 3.3	72.2 ± 3.5
Height (cm)	166.5 ± 8.3	164.2 ± 5.9
Weight (kg)	65.1 ± 15.4	63.7 ± 11.8
Total number of teeth remaining	23.1 ± 1.5	23.9 ± 1.7
Masseter muscle thickness	6.78 ± 0.56	7.09 ± 0.62
Maximum occlusal force	254.85 ± 6.32	260.17 ± 9.72
Stimulation intensity (mA)	7.3 ± 2.5	NA

**Table 3:** Changes of masseter muscle thickness and maximum occlusal force in parameters before and after treatment.

	Experimental Group			Control Group			Intergroup
	Before	After	*p-*Value	Before	After	*p-*Value	*p*-Values
MMT (mm)	6.78 ± 0.56	8.76 ± 0.60	<0.001 *	7.09 ± 0.62	7.34 ± 0.71	0.009 *	<0.001 ^†^
MOF (Newton)	254.85 ± 6.32	287.62 ± 8.11	<0.001 *	260.17 ± 9.72	277.33 ± 6.84	0.002 *	0.008 ^†^

Mean ± standard deviation, MMT: Masseter muscle thickness, MOF: Maximum occlusal force * *p* < 0.05 by paired t-test, ^†^ *p* < 0.05 by independent t-test.

**Table 4:** Comparison of amount of change in each group.

	Experimental Group	Control Group	*p-*Value	Cohen’s D (Interpretation)
Δ MMT (mm)	1.97 ± 0.85	0.25 ± 0.29	<0.001	0.6 (moderate effect)
Δ MOF (Newton)	32.77 ± 8.02	17.17 ± 11.01	<0.001	0.8 (large effect)

Mean ± standard deviation, MMT: Masseter muscle thickness, MOF: Maximum occlusal force.

## References

[1] Ashiga H., Takei E., Magara J., Takeishi R., Tsujimura T., Nagoya K., Inoue M. (2019). Effect of attention on chewing and swallowing behaviors in healthy humans. Sci. Rep..

[2] Ohira A., Ono Y., Yano N., Takagi Y. (2012). The effect of chewing exercise in preschool children on maximum bite force and masticatory performance. Int. J. Paediatr. Dent..

[3] Ide Y. (2010). Structural characterictics of the swallowing organ. Jpn. J. Rehabil. Med..

[4] Fukamizu K., Kodama M., Moriya Y., Yokoyama M., Murata Y. (1972). The biting forces and the electromyographic analyses of the temporalis and masseter muscles in the dentulous mandibular positions. Nihon Hotetsu Shika Gakkai Zasshi.

[5] Borzuola R., Giombini A., Torre G., Campi S., Albo E., Bravi M., Borrione P., Fossati C., Macaluso A. (2020). Central and Peripheral Neuromuscular Adaptations to Ageing. J. Clin. Med..

[6] Okada K., Enoki H., Izawa S., Iguchi A., Kuzuya M. (2010). Association between masticatory performance and anthropometric measurements and nutritional status in the elderly. Geriatr. Gerontol. Int..

[7] Sheiham A., Steele J.G., Marcenes W., Lowe C., Finch S., Bates C.J., Prentice A., Walls A.W. (2001). The relationship among dental status, nutrient intake, and nutritional status in older people. J. Dent. Res..

[8] Reber E., Gomes F., Dähn I.A., Vasiloglou M.F., Stanga Z. (2019). Management of Dehydration in Patients Suffering Swallowing Difficulties. J. Clin. Med..

[9] Roh H.T., Cho S.Y., So W.Y. (2020). A Cross-Sectional Study Evaluating the Effects of Resistance Exercise on Inflammation and Neurotrophic Factors in Elderly Women with Obesity. J. Clin. Med..

[10] Park J.S., Jung Y.J., Kim H.H., Lee G. (2019). A Novel Method Using Kinesiology Taping for the Activation of Suprahyoid Muscles in Healthy Adults: A Preliminary Research. Dysphagia.

[11] Yano J., Yamamoto-Shimizu S., Yokoyama T., Kumakura I., Hanayama K., Tsubahara A. (2020). Effects of Tongue-Strengthening Exercise on the Geniohyoid Muscle in Young Healthy Adults. Dysphagia.

[12] Shirai M., Kawai N., Hichijo N., Watanabe M., Mori H., Mitsui S.N., Yasue A., Tanaka E. (2018). Effects of gum chewing exercise on maximum bite force according to facial morphology. Clin. Exp. Dent. Res..

[13] Nakagawa K., Matsuo K., Takagi D., Morita Y., Ooka T., Hironaka S., Mukai Y. (2017). Effects of gum chewing exercises on saliva secretion and occlusal force in community-dwelling elderly individuals: A pilot study. Geriatr. Gerontol. Int..

[14] Ludlow C.L., Humbert I., Saxon K., Poletto C., Sonies B., Crujido L. (2007). Effects of surface electrical stimulation both at rest and during swallowing in chronic pharyngeal dysphagia. Dysphagia.

[15] Suiter D.M., Leder S.B., Ruark J.L. (2006). Effects of neuromuscular electrical stimulation on submental muscle activity. Dysphagia.

[16] Ferreira A.P., Costa D.R., Oliveira A.I., Carvalho E.A., Conti P.C., Costa Y.M., Bonjardim L.R. (2017). Short-term transcutaneous electrical nerve stimulation reduces pain and improves the masticatory muscle activity in temporomandibular disorder patients: A randomized controlled trial. J. Appl. Oral Sci..

[17] Wang J.S., Lee J.H., Kim N.J. (2015). Effects of neuromuscular electrical stimulation on masticatory muscles in elderly stroke patients. J. Phys. Ther. Sci..

[18] Lee K.W., Kim S.B., Lee J.H., Lee S.J., Park J.G., Jang K.W. (2019). Effects of Neuromuscular Electrical Stimulation for Masseter Muscle on Oral Dysfunction After Stroke. Ann. Rehabil. Med..

[19] Umay E.K., Yaylaci A., Saylam G., Gundogdu I., Gurcay E., Akcapinar D., Kirac Z. (2017). The effect of sensory level electrical stimulation of the masseter muscle in early stroke patients with dysphagia: A randomized controlled study. Neurol. India.

[20] Byeon H. (2020). Combined Effects of NMES and Mendelsohn Maneuver on the Swallowing Function and Swallowing-Quality of Life of Patients with Stroke-Induced Sub-Acute Swallowing Disorders. Biomedicines.

[21] Jung S.H., Kim Y.A., Hwang N.K., Park J.S., Kim Y. (2018). Effects of neuromuscular electrical stimulation in combination with saliva or dry swallowing in stroke patients with dysphagia. J. Korean Dysphagia Soc..

[22] Sproson L., Pownall S., Enderby P., Freeman J. (2018). Combined electrical stimulation and exercise for swallow rehabilitation post-stroke: A pilot randomized control trial. Int. J. Lang. Commun. Disord..

[23] Rowlerson A., Raoul G., Daniel Y., Close J., Maurage C.A., Ferri J., Sciote J.J. (2005). Fiber-type differences in masseter muscle associated with different facial morphologies. Am. J. Orthod. Dentofac. Orthop..

[24] Chang M.Y., Lee G., Jung Y.J., Park J.S. (2020). Effect of Neuromuscular Electrical Stimulation on Masseter Muscle Thickness and Maximal Bite Force among Healthy Community-Dwelling Persons Aged 65 Years and Older: A Randomized, Double Blind, Placebo-Controlled Study. Int. J. Environ. Res. Public Health.

[25] Trawitzki L.V., Borges C.G., Giglio L.D., Silva J.B. (2011). Tongue strength of healthy young adults. J. Oral Rehabil..

[26] Galo R., Vitti M., da Glória Chiarello Mattos M., Regalo S.C. (2007). Masticatory muscular activation in elderly individuals during chewing. Gerodontology.

[27] Farina D., Merletti R., Enoka R.M. (2014). The extraction of neural strategies from the surface EMG: An update. J. Appl. Physiol..

[28] Rau G., Schulte E., Disselhorst-Klug C. (2004). From cell to movement: To what answers does EMG really contribute?. J. Electromyogr. Kinesiol..

[29] Huang K.L., Liu T.Y., Huang Y.C., Leong C.P., Lin W.C., Pong Y.P. (2014). Functional outcome in acute stroke patients with oropharyngeal Dysphagia after swallowing therapy. J. Stroke Cerebrovasc. Dis..

[30] Shaw G.Y., Sechtem P.R., Searl J., Keller K., Rawi T.A., Dowdy E. (2007). Transcutaneous neuromuscular electrical stimulation (VitalStim) curative therapy for severe dysphagia: Myth or reality?. Ann. Otol. Rhinol. Laryngol..

[31] Miljkovic N., Lim J.Y., Miljkovic I., Frontera W.R. (2015). Aging of skeletal muscle fibers. Ann. Rehabil. Med..

[32] Freed M.L., Freed L., Chatburn R.L., Christian M. (2001). Electrical stimulation for swallowing disorders caused by stroke. Respir. Care.

[33] Permsirivanich W., Tipchatyotin S., Wongchai M., Leelamanit V., Setthawatcharawanich S., Sathirapanya P., Phabphal K., Juntawises U., Boonmeeprakob A. (2009). Comparing the effects of rehabilitation swallowing therapy vs. neuromuscular electrical stimulation therapy among stroke patients with persistent pharyngeal dysphagia: A randomized controlled study. J. Med. Assoc. Thail..

[34] Carnaby-Mann G.D., Crary M.A. (2008). Adjunctive neuromuscular electrical stimulation for treatment-refractory dysphagia. Ann. Otol. Rhinol. Laryngol..

[35] Blumenfeld L., Hahn Y., Lepage A., Leonard R., Belafsky P.C. (2006). Transcutaneous electrical stimulation versus traditional dysphagia therapy: A nonconcurrent cohort study. Otolaryngol. Head Neck. Surg..

[36] Tan C., Liu Y., Li W., Liu J., Chen L. (2013). Transcutaneous neuromuscular electrical stimulation can improve swallowing function in patients with dysphagia caused by non-stroke diseases: A meta-analysis. J. Oral Rehabil..

